# Predicting Plant Performance Under Simultaneously Changing Environmental Conditions—The Interplay Between Temperature, Light, and Internode Growth

**DOI:** 10.3389/fpls.2015.01130

**Published:** 2015-12-21

**Authors:** Katrin Kahlen, Tsu-Wei Chen

**Affiliations:** ^1^Department of Vegetable Crops, Geisenheim UniversityGeisenheim, Germany; ^2^Institute of Horticultural Production Systems, Faculty of Natural Sciences, Leibniz Universität HannoverHannover, Germany

**Keywords:** plant development, plant growth, morphogenesis, virtual plant, cucumber, Arrhenius, modeling

## Abstract

Plant performance is significantly influenced by prevailing light and temperature conditions during plant growth and development. For plants exposed to natural fluctuations in abiotic environmental conditions it is however laborious and cumbersome to experimentally assign any contribution of individual environmental factors to plant responses. This study aimed at analyzing the interplay between light, temperature and internode growth based on model approaches. We extended the light-sensitive virtual plant model *L-Cucumber* by implementing a common Arrhenius function for appearance rates, growth rates, and growth durations. For two greenhouse experiments, the temperature-sensitive model approach resulted in a precise prediction of cucumber mean internode lengths and number of internodes, as well as in accurately predicted patterns of individual internode lengths along the main stem. In addition, a system's analysis revealed that environmental data averaged over the experimental period were not necessarily related to internode performance. Finally, the need for a species-specific parameterization of the temperature response function and related aspects in modeling temperature effects on plant development and growth is discussed.

## Introduction

Accurate prediction of plant performance under changing environmental conditions is a crucial prerequisite for advancements in various frontiers in plant research. To this end, an assessment of impacts of possible climate change on plant productivity and food security (Asseng et al., 2013) or a development of adapted crop production systems that may capture the environmental challenges of future growing seasons (e.g., He et al., 2015; O'Leary et al., 2015) are of particular interest. However, plant performance is significantly influenced e.g., by light, temperature, vapor pressure deficit, or soil water content. These environmental factors act simultaneously on signal pathways and physiological networks. Moreover, plant responses to these factors depend on the developmental stage and in particular on location of the resource harvesting organs in space and time (e.g., Kahlen and Stützel, 2011b; Wiechers et al., 2011; Malekpoor Mansoorkhani et al., 2014). For example, light harvest of a plant depends both on respective architectural traits of the plant itself as well as of its neighbors (Vandenbussche et al., 2005; Long et al., 2006). An important architectural trait for the light distribution in the canopy is the internode length, with longer internodes being e.g., beneficial for whole-plant light distribution and biomass production (e.g., Sarlikioti et al., 2011).

Internode growth and development of cucumber plants depend on light quantity (photosynthetically active radiation, PAR) and quality of signals (red to far-red ratio, R:FR). Therefore, natural light fluctuations may result in widespread patterns of final internode lengths along the main stem. A modeling study showed that it is possible to predict the internode performance of cucumber plants under fluctuating light conditions in greenhouses (Kahlen and Stützel, 2011a). However, this model is only valid for a constant temperature of 20°C. Since temperature conditions during plant growth and development significantly influence plant architecture (e.g., Poiré et al., 2010; Wigge, 2013), any effects of variable temperatures on internode performance should be taken into account in modeling approaches. In a recent simulation study, Chen et al. (2014) developed a temperature-sensitive growth model for tomatoes using different temperature response functions for elongation rates and durations of developmental processes. This model accurately predicted differences in tomato plant height for two different temperatures, but predictions for individual internode lengths were less precise. Moreover, the model by Chen et al. (2014) differs from the recent finding that temperature responses of organ elongation and durations of developmental cycles follow a single Arrhenius-type response curve after normalization with a reference temperature (Parent et al., 2010; Parent and Tardieu, 2012).

This work aimed at disentangling light and temperature effects on final internode lengths based on a model approach. We extended the light-sensitive virtual plant model *L-Cucumber* (Kahlen and Stützel, 2011a) by implementing temperature responsiveness of developmental processes (e.g., elongation rates and durations) as proposed by Parent and Tardieu (2012). We hypothesized that integrating a common temperature-response function will improve prediction qualities compared to the original *L-Cucumber* model. Simulation scenarios were run using environmental data of two greenhouse experiments. Accurate predictions of final internode lengths would subsequently allow us to analyze contributions of light and temperature to internode performance by the comparing simulation outputs of both the temperature-sensitive and -insensitive models.

## Materials and methods

### The original *L-Cucumber* model

The dynamic virtual plant model *L-Cucumber* predicts growth and development of greenhouse grown cucumber plants under various light conditions and canopy architectures (Kahlen and Stützel, 2011a). Production of dry matter and growth of the shoot are dependent on the local light conditions of each individual leaf within the canopy, which are provided by a coupled light model (Kahlen and Stützel, 2011a). Therefore, *L-Cucumber* is a typical functional-structural plant model. Virtual leaves have optical properties similar to those of cucumber leaves with 6% reflectance and 7% transmittance of incident PAR including red light (R) and 38% reflectance and 45% transmittance for far red light (FR; Kahlen et al., 2008). Thus, due to the complex interactions of the light passing through the canopy, local R:FR ratios are emerging properties of the model. Model predictions of internode lengths depend on PAR and local R:FR ratios.

In the original model, final internode length, FIL, depends on both, light quantity and light quality [Kahlen and Stützel, 2011a; Equation (MA2)]:

(1) $$\begin{array}{l} \text{FIL}\left(\text{PAR},\text{R}:\text{FR}\right)=\;13.4\;-\;0.014\cdot \text{ PAR }+\;f\left(\text{R}:\text{FR}\right) \end{array}$$

PAR is the mean PAR (μmol m^−2^ s^−1^) above the canopy of 4 days starting 6 days before the internode has reached its maximum growth rate (in the following referred to as PAR4d). R:FR is the mean value of the R:FR ratio at the whole stem in the same period as the PAR signal and the R:FR at the internode at its maximum growth rate. The function *f* (R:FR) represents a stepwise linear response of the final internode to local light quality (Kahlen and Stützel, 2011a; their Figure 7).

All experiments used for parameterization and evaluation of the original model approach were conducted using greenhouse set point temperatures for day/night of 20°C/16°C and 24°C as a threshold for opening of greenhouse ventilation. Therefore, the basic assumption for the following approach is that the response functions of *L-Cucumber* are valid for an average daily temperature of 20°C. Considering a base temperature of 10°C, the simulated growth duration starting at an internode length of 3 cm until 95% of the final length is reached takes 42°Cd (Kahlen and Stützel, 2011a). Thus, the elongation duration at 20°C, *D*_*e*_(20°C), is

(2a) $$\begin{array}{l} D_{e}\left(20^{{}^{\circ}}\text{C}\right)\;=\;4.2\text{ d} \end{array}$$

The appearance rate of cucumber internodes at 20°C is 0.7 d^−1^ (Kahlen and Stützel, 2011a). Therefore, the reciprocal of this value is equivalent to the duration between the appearance of two successive leaves at the reference temperature of 20°C, *D*_*a*_(20°C):

(2b) $$\begin{array}{l} D_{a}\left(20^{{}^{\circ}}\text{C}\right)\;=\;1.4\text{ d} \end{array}$$

### Temperature responses of developmental processes

**Figure 1:**
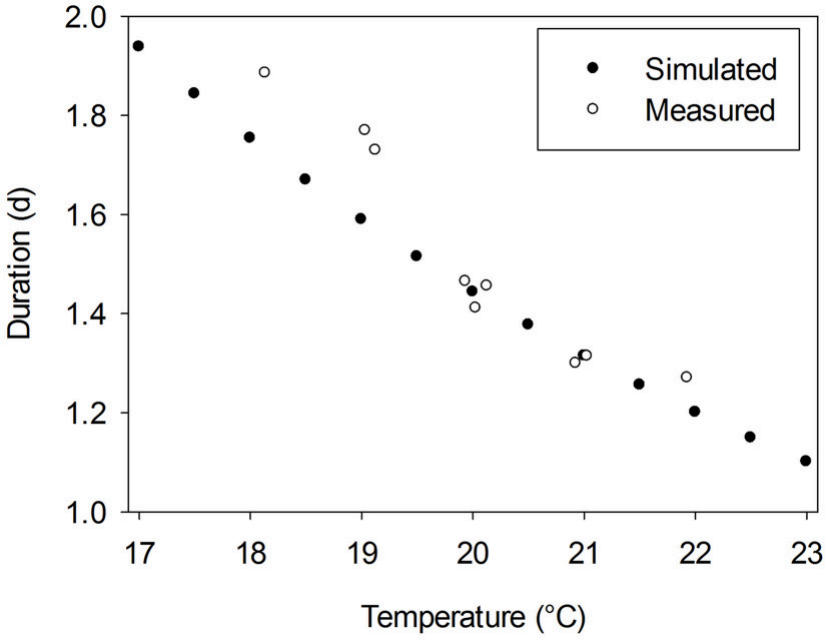
**Measured duration between the appearance of two successive leaves for greenhouse grown cucumber plants, cv. Aramon, derived from data of Papadopoulos and Hao (2000) vs. estimated durations using an average temperature response function, *F_av_*(T), which is based on the approach of Parent and Tardieu (2012)**.

The study of Parent and Tardieu (2012) provides a guideline for implementing temperature-responses into models for developmental processes. A temperature-modulated elongation rate can be estimated by its reference value at 20°C multiplied by the value of the crop-specific Arrhenius function for the considered temperature. A temperature-modulated duration of a phase at a given temperature equals its reference value at 20°C divided by the value for the considered temperature. Here, the Arrhenius function in relation to temperature (*T*) equals:

(3) $$\begin{array}{l} F\left(T\right)=\frac{ATe^{\left(-△H_{A}∕RT\right)}}{1+{\left[e^{\left(-△H_{A}∕RT\right)}\right]}^{\alpha \left(1-T∕T_{0}\right)}} \end{array}$$

where *R* is the gas constant (8.314 J mol^−1^ K^−1^), Δ*H*_*A*_ is the enthalpy of activation (J mol^−1^) and *T*_0_ is the temperature at which half of the system is in an active state (K). Since α is a dimensionless constant, which is assumed to equal 3.5 and *A* is a scaling coefficient depending on Δ*H*_*A*_, *T*_0_, and α (for more details see Parent and Tardieu, 2012; their Supplementary Table 2), a full crop-specific parameterization of *F(T)* requires data for Δ*H*_*A*_ and *T*_0_. Parent and Tardieu (2012) presented the corresponding values for 17 crop species and *Arabidopsis*. However, a cucumber-specific parameterization of the Arrhenius function is not available. Thus, we used the data of these 18 plant species to estimate average parameters. The average activation enthalpy, Δ*H*_*A*_ (69350 J mol^−1^) and the average temperature at which half of the system is in an active state, *T*_0_ (305 K) were used as input data for an average temperature response function of all 18 species, *F*_av_(T). From the study of Papadopoulos and Hao (2000) we derived durations between the appearance of two successive leaves for greenhouse grown cucumber plants cultivated under temperature conditions in the range from 18 to 22°C. Predicted durations using *F*_av_(T) matched derived data under temperatures above 20°C, but slightly overestimated derived durations under lower temperatures (Figure 1). Since typical greenhouse temperature is above 20°C, we assumed that *F*_av_(T) is an appropriate initial estimator for a cucumber-specific temperature response function.

Therefore, the function *F*_av_(*T*) was used to extend the corresponding response functions in *L-Cucumber*. For internode elongation rates, the model assumes the same temperature-sensitive period as for the signals of light quantity. Thus, the elongation rate of an internode, ER(*t*) at day *t* (cm), is estimated using the following response function:

(4) $$\begin{array}{r} \text{ER}\left(t\right)\;=\;k\;\cdot \;\left({\text{FIL}}_{T}\left(\text{T},\text{ PAR},\text{ R}:\text{FR}\right)\;-\text{ IL}\left(t\right)\right)\\ =\;k\;\cdot \;\left(F_{\text{av}}\left(\text{T}\right)\;\cdot \text{ FIL}\left(\text{PAR},\text{R}:\text{FR}\right)\;-\text{ IL}\left(t\right)\right) \end{array}$$

*F*_av_(T) is the average temperature response function for temperature T in the same period as the PAR signal. This is a mean temperature of 4 days starting 6 days before the internode has reached its maximum growth rate (in the following referred to as T4d). The timing of PAR and R:FR are the same as in the original model (Equation 1). IL(*t*) is the internode length at day *t* and *k* is 50% (taken from Kahlen and Stützel, 2011a).

The elongation duration for internode growth, *D*_*e*_(T) (d), is determined at simulated internode appearance:

(5) $$\begin{array}{l} {\text{D}}_{e}\left(\text{T}\right)\;=\;{\text{D}}_{\text{e}20}\;∕\;F_{av}\left(\text{T}\right) \end{array}$$

with the same temperature signal as for the elongation rate.

In agreement with the above-mentioned concept, temperature-responsiveness of the appearance rate is calculated via:

(6) $$\begin{array}{l} {\text{D}}_{a}\left(\text{T}\right)\;=\;{\text{D}}_{\text{a}20}\;∕\;F_{av}\left(\text{T}\right) \end{array}$$

In this case, temperature T at each day during development is used. The appearance rate is the reciprocal of *D*_*a*_(T).

### Experiments

Two experiments (E1 and E2) were carried out in an experimental greenhouse at the Department of Vegetable Crops, Geisenheim University, Germany (lat. 49° 59′ N, long. 7° 58′ E). Except for the growing medium (here “Profisubstrat spezial” SP T HF from *Patzer*, Germany), plant nursery and cultivation were the same as described by Kahlen and Stützel (2007). Cucumber plants (*Cucumis sativus* L., cv. Aramon) were transplanted at the two-leaf stage. Plant to plant distance within the row were 30 cm resulting in an overall plant density of 1.9 plants m^−2^. Set point temperatures in the greenhouse for day and night were 20/16°C. Ventilation opened at 24°C. See Supplementary Table 1 for scheduling details of the experiments.

Daily global radiation data (μmol m^−2^ s^−1^) were recorded by a weather station located next to the greenhouse. Due to the properties of the greenhouse construction, ca. 50% of outside PAR was available inside the greenhouse. Daily PAR for model input considers the day length. Temperature conditions in a canopy can be quite heterogeneous (e.g., Chelle and Cellier, 2009). In our study, all temperature-related parameters characterize physiological processes occurring at the meristem and at young growing organs of unstressed plants. In our experiments and model approaches, the growing parts of plants were located at the top of the canopy. Therefore, we assumed that air temperature could be a good indicator temperature for our analysis. Temperature in the middle of the greenhouse was recorded every minute. Daily mean temperatures were used the simulations.

The lengths of internodes at rank 5 to the top of the plant were measured using a ruler at 4 measurement dates (in E1 at day 132, 137, 144, and 150 of year; in E2 at day 179, 182, 189, and 192 of year). Internodes at lower ranks were not considered, because they had been initiated before the plants were transplanted to the experimental greenhouse. To prevent plant damages caused by the measurement, minimal internode lengths were 3 cm. The number of internodes with at least minimal length was used to determine appearance rates. For further data analysis, the average data of 12 plants were used.

### Simulations

To assess the prediction quality of the temperature-sensitive model for internode performance under changing temperature and light conditions, simulations with the extended *L-Cucumber* model (MA-T) were run. All simulations were also run with a constant mean temperature of 20°C, which corresponds to the original *L-Cucumber* model (MA-20) and thus a temperature-insensitive model approach. These simulations allow us to assess the effects of temperature on internode performance and resulting internode lengths: Based on the differences in average final internode lengths and in the number of internodes simulated temperature effects were separated from simulated light effects.

The virtual scenes corresponded to the individual canopy set-up of the experiments (e.g., distances between plants, training, and pruning system). Model input was a data set of measured daily mean PAR and daily mean temperature during the cultivation periods of the above-described experiments. The coupled light model simulated a hemispherical approximation of the sky at the location of the experiments and was set up for the path of the sun on a fixed day of the year. Here, day of year equals 140 for E1 and 180 for E2, respectively. The local light quality conditions (R:FR) emerges from the interaction of canopy architecture and optical light properties of the virtual canopy. Further details on the virtual canopy can be found in Kahlen and Stützel (2011a).

For both experiment, individual simulations were run for five slightly different initial plant orientations with five replications. Simulations started with the appearance of the first true leaf and internode at time step 2. By this time, the first leaf has already an area of 50 cm^2^ and the internode a length of 3 cm. Each time step comprised 1 d to consider daily changes in light and temperature. For assessing the quality of the models, only simulated data of internodes at rank 5 and higher were considered (see also Kahlen and Stützel, 2011a). Corresponding to the measurement dates, simulation steps 11, 16, 23, and 29 for E1 and 12, 15, 22, and 25 for E2 were used for subsequent data analysis.

### Statistics and analysis of environmental conditions

Measured and simulated data were compared using ANOVA, if normality tests and tests for equal variance were passed, or else Kruskal-Wallis with *P* = 0.05, using R programming language [R version 3.1.0 by R Core Team (2014)]. Means or medians and standard deviations were also calculated in R. To assess the prediction quality of the models, we additionally provided data on root mean square deviation (RMSD), mean deviation (bias) and systematic prediction error (SPE) (Kobayashi and Salam, 2000) for final internode length (FIL). The Pearson correlation coefficient, *r*, was used to indicate whether radiation and temperature in a given experimental phase were associated. The coefficient of variation, *CV*, shows the standard deviation (SD) as a percentage of the mean. We used the *CV* in order to compare data with different orders or different units, such as PAR and temperature.

PAR and temperature contributions were assigned to average and individual internode lengths based on (i) model analysis and (ii) model comparisons (MA-T vs. MA-20). The PAR-sensitive part of the model (the first linear term in Equation 1) was used to estimate the PAR-dependent length contribution. Temperature effects on internode performance were estimated from rank-specific temperatures and the corresponding Arrhenius factors.

## Results

**Figure 2:**
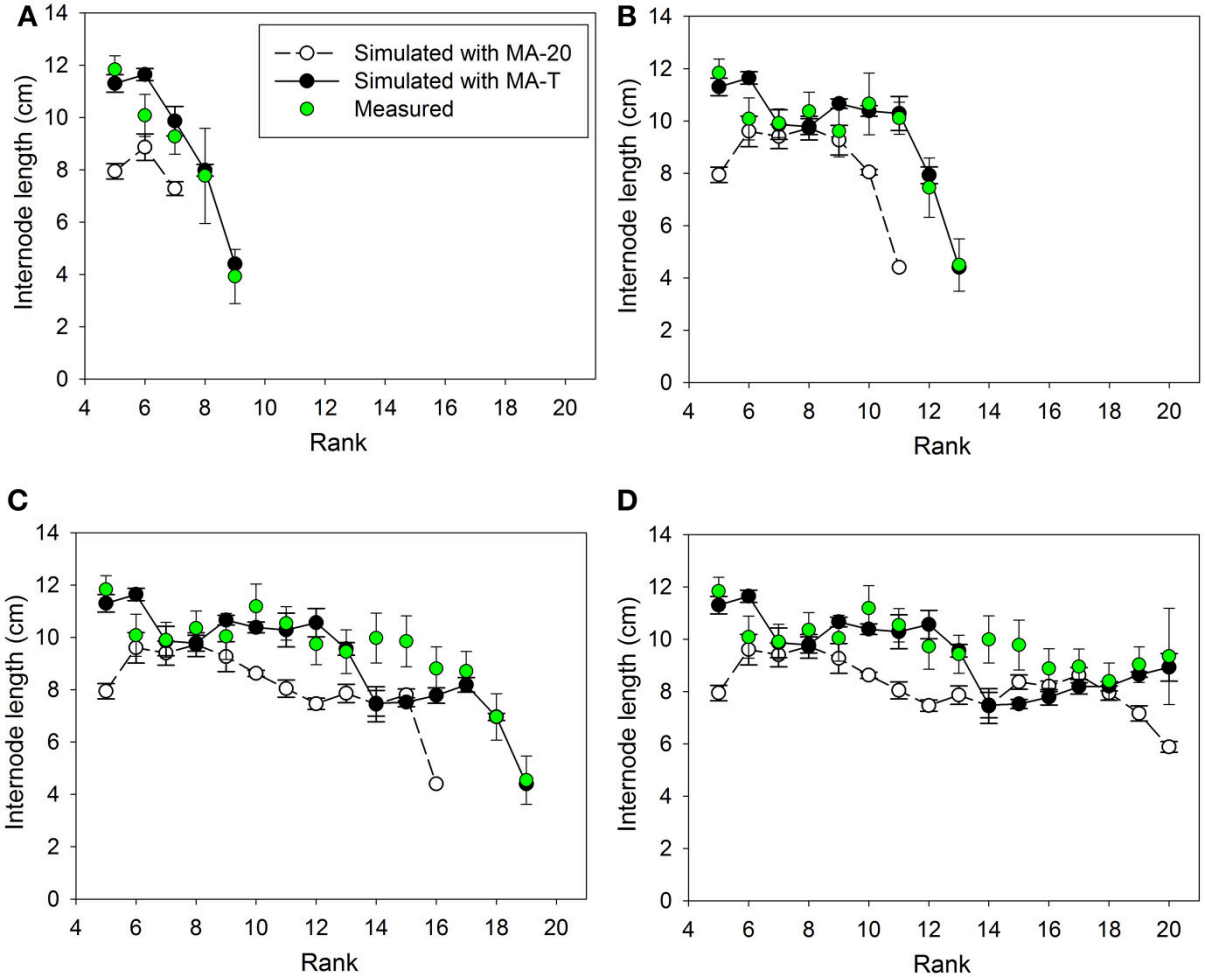
**Measured and simulated patterns of internode lengths along the main stem of greenhouse grown cucumber plants for rank 5 to the top of the plant for measured and simulated organ sizes**. The measured data were obtained in Experiment 1 (E1) and the simulated patterns resulted from the temperature-insensitive model (MA-20) and the temperature-sensitive model (MA-T). Measured (*n* = 12) and simulated (*n* = 5) data were from day of year **(A)** 132, **(B)** 137, **(C)** 144, and **(D)** 150. Vertical bars represent standard deviations. Straight lines, which connect symbols, are shown to visualize the pattern of simulated internode lengths along the main stem.

**Figure 3:**
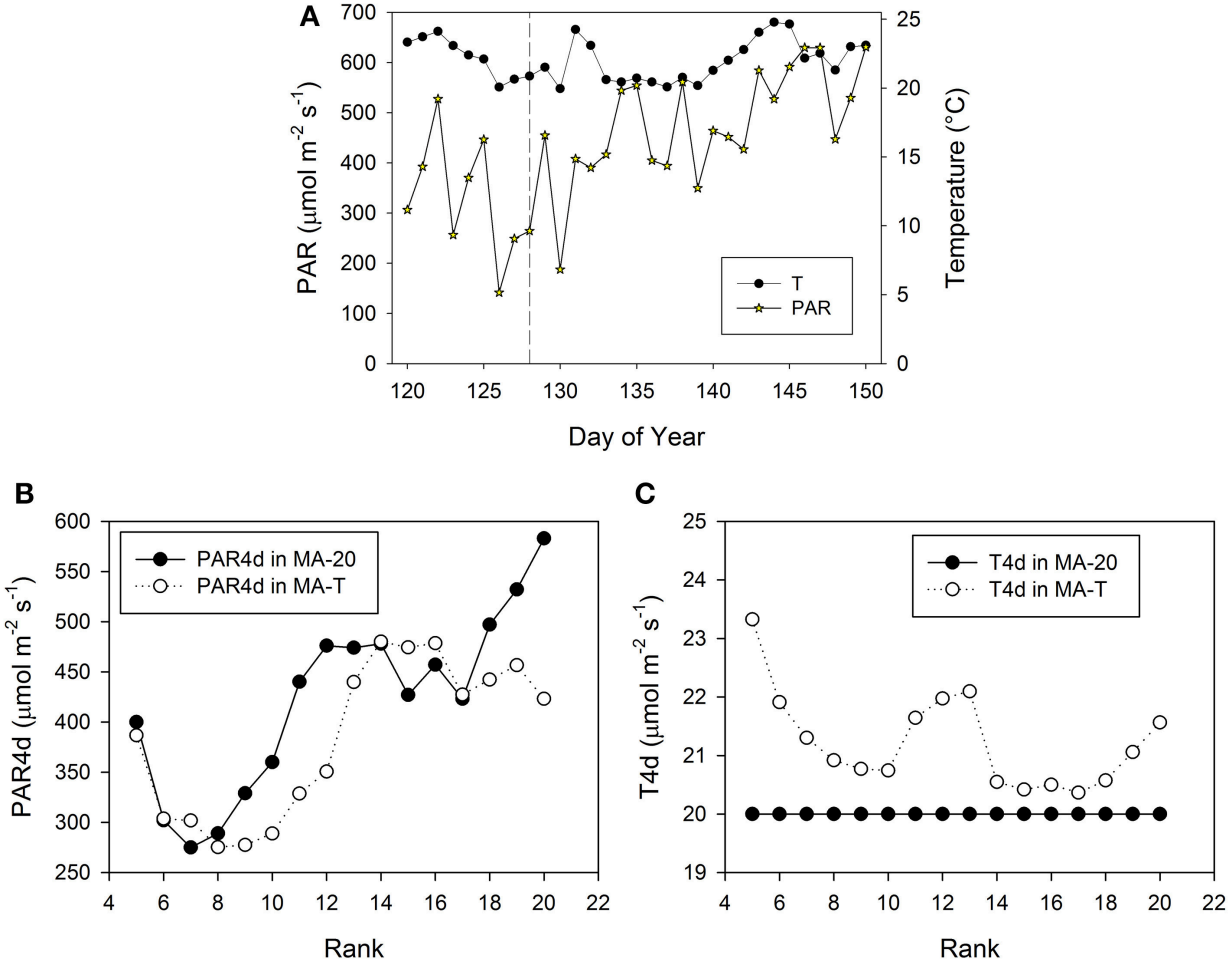
**Measured climatic data of photosynthetic active radiation, PAR (μmol m^**−2**^ s^**−1**^) and mean daily temperature (°C, 24 h) inside the greenhouse in Experiment E1 (A)**. The vertical dashed line indicates the day, when the internodes at rank 5 had a length of ca. 3 cm. PAR4d (μmol m^−2^ s^−1^) is the mean PAR of 4 days starting 6 days before the internode has reached its maximum growth rate **(B)**. T4d (°C) is the corresponding temperature data **(C)**.

**Figure 4:**
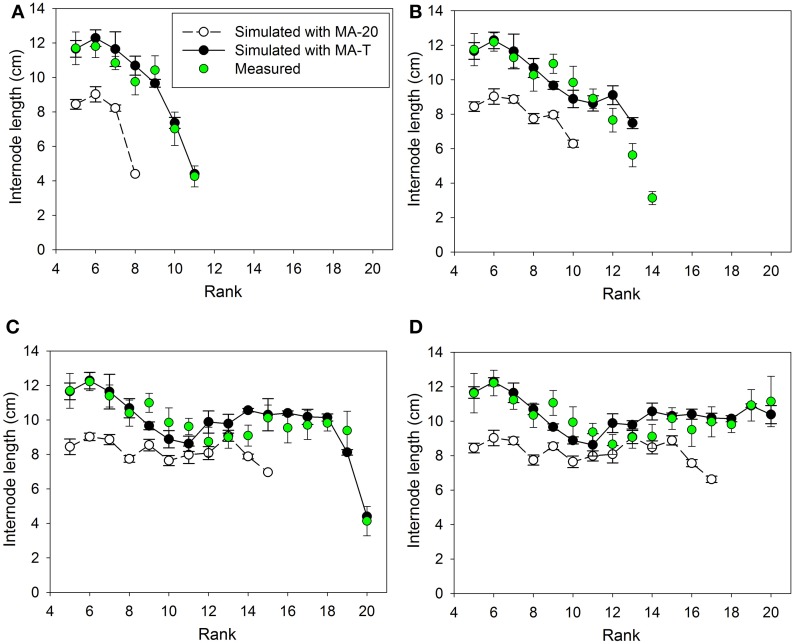
**Measured and simulated patterns of internode lengths along the main stem of greenhouse grown cucumber plants for rank 5 to the top of the plant for measured and simulated organ sizes**. The measured data were obtained in Experiment 2 (E2) and the simulated patterns resulted from the temperature-insensitive model (MA-20) and the temperature-sensitive model (MA-T). Measured (*n* = 12) and simulated (*n* = 5) data were from day of year **(A)** 179, **(B)** 182, **(C)** 189, and **(D)** 192. Vertical bars represent standard deviations. Straight lines, which connect symbols, are shown to visualize the pattern of simulated internode lengths along the main stem.

**Figure 5:**
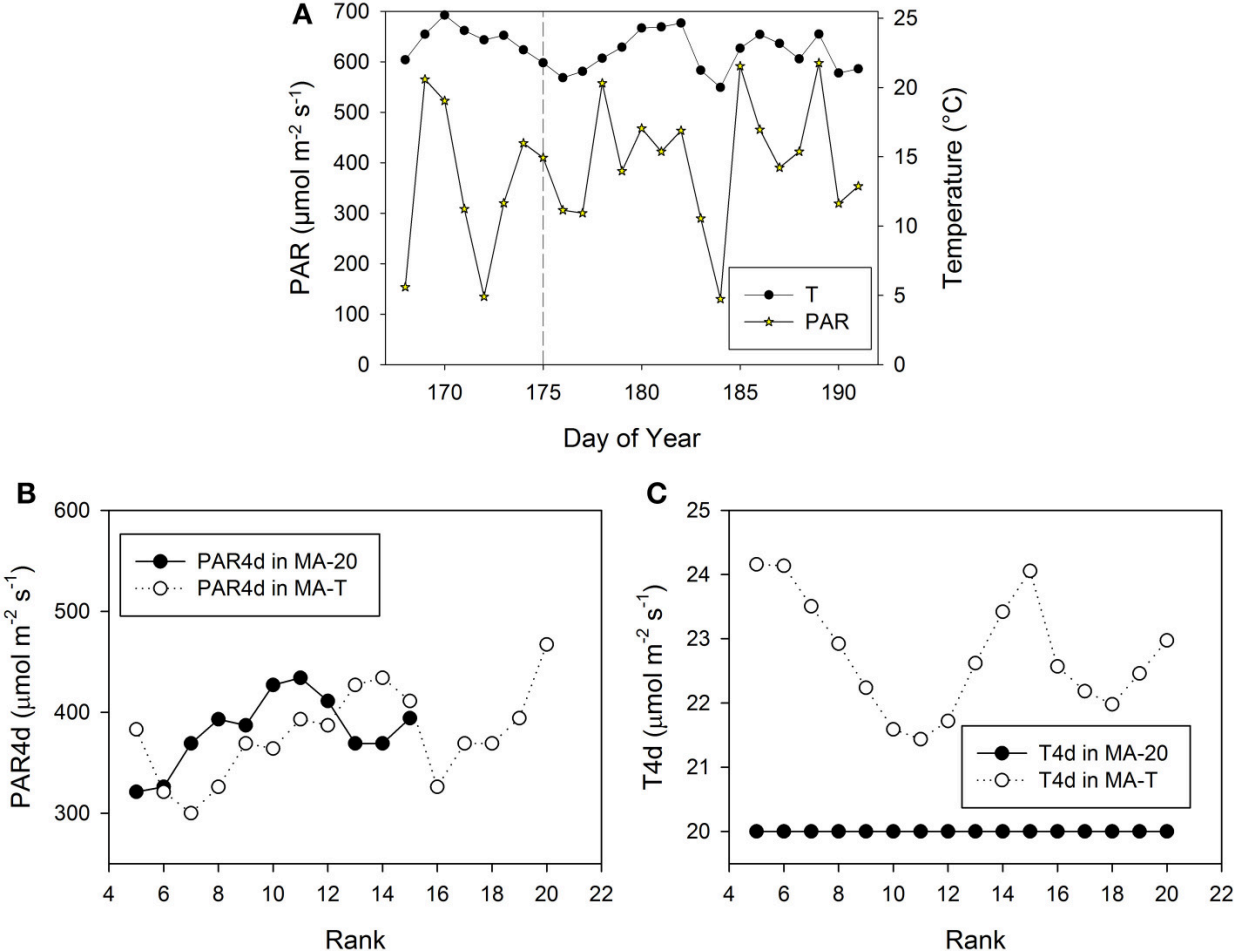
**Measured climatic data of photosynthetic active radiation, PAR (μmol m^**−2**^ s^**−1**^) and mean daily temperature (°C, 24 h) inside the greenhouse in Experiment E2 (A)**. The vertical dashed line indicates the day, when the internodes at rank 5 had a length of ca. 3 cm. PAR4d (μmol m^−2^ s^−1^) is the mean PAR of four days starting six days before the internode has reached its maximum growth rate **(B)**. T4d (°C) is the corresponding temperature data **(C)**.

Measured internode lengths and corresponding model predictions using MA-T, the temperature-sensitive model approach, and MA-20, the temperature-insensitive approach, for plants grown in Experiments E1 and E2 are shown in Figures 2, **4**, respectively. In addition, Figures 3A, **5A** present the pattern of daily mean PAR and daily mean temperatures measured in the experiments. The simulation scenarios based on MA-T used this climatic data as input, whereas MA-20 used measured radiation data, but input temperatures equaled 20°C for the whole simulation period. Except for these differences in input temperatures, there were no differences between the two model approaches. Using MA-T, predicted patterns of internode lengths along the main stem were similar to measured patterns for both experiments (Figures 2, 4).

### Quality of model predictions—mean lengths and number of internodes

Using MA-T, the data agreed for both, numbers of FILs along the main stem of the plants and mean final internode lengths (Table 1). Over all internodes, which had reached their final sizes, the variations of simulated and measured mean FIL were in good agreement for all simulation steps (Table 1). On the other hand, the prediction quality of MA-20 was significantly lower for both experiments. RMSDs and biases were on average more than 1 cm and 2 cm larger than with MA-T for E1 and E2, respectively. For E2, e.g., the biases in FILs were even almost three times larger than with MA-T (Table 1). The corresponding systematic prediction errors were above 75%. In contrast, using MA-T, they decreased with time and were almost negligible at the last time step. For E2, predicted lengths of the internode using MA-T and MA-20 were different at any rank (>0.7 cm, Figure 4D), whereas for E1 internodes at a few ranks (ranks 7, 8, 14, 15) had the same lengths for both model approaches (Figure 2D). For E1, MA-20 resulted in an underestimation of the number of internodes by 2 on all measurement days, however, for E2 an increasing underestimation from 2 to 5 internodes was observed (Table 1).

**Table 1:** **Statistical analysis of measured and simulated data for greenhouse grown cucumber plants grown in Experiments 1 and 2**.

**Experiment**	**DOY/Step**	**Data type**	**RMSD (cm)**	**Bias (cm)**	**SPE %**	**Number**	**Mean length (cm)**	**Coefficient of variation %**
1	132/11	Measured				3	10.4 ± 1.1	10
		MA-20	3.9	3.9	100	1	7.9	
		MA-T	1.0	−0.5	29	3	10.9 ± 0.8	7
	137/16	Measured				7	10.4 ± 0.7	7
		MA-20	1.8	1.2	42	5	9.2 ± 0.6	7
		MA-T	0.8	−0.2	6	7	10.6 ± 0.6	6
	144/23	Measured				12	10.1 ± 0.7	7
		MA-20	2.1	1.8	72	10	8.5 ± 0.9	10
		MA-T	1.2	0.4	12	13	9.6 ± 1.4	14
	150/29	Measured				16	9.8 ± 0.9	9
		MA-20	1.8	1.5	65	14	8.5 ± 0.7	9
		MA-T	1.1	0.4	15	16	9.4 ± 1.3	14
2	179/12	Measured				4	11.0 ± 0.8	7
		MA-20	3.0	3.0	99	2	8.7 ± 0.3	3
		MA-T	0.7	−0.5	68	4	11.6 ± 0.6	5
	182/15	Measured				7	10.7 ± 1.1	10
		MA-20	2.8	2.9	107	5	8.4 ± 0.5	6
		MA-T	0.6	0.2	14	7	10.5 ± 1.3	13
	189/22	Measured				14	10.2 ± 1.0	10
		MA-20	2.3	2.1	79	9	8.4 ± 0.5	6
		MA-T	0.8	−0.2	5	14	10.3 ± 1.0	10
	192/25	Measured				16	10.3 ± 1.0	10
		MA-20	2.1	1.8	75	11	8.4 ± 0.5	6
		MA-T	0.8	−0.1	2	16	10.4 ± 0.9	9

*The data refers to the lengths of internodes (above rank 4), which had reached their final lengths at the day of measurement (DOY) or, in case of the simulation results, at the corresponding simulation step. The simulation approach is either sensitive light conditions only (PAR and RFR in MA-20) or also temperature-sensitive (MA-T). Statistical means, such as the root mean squared deviation (RMSD), bias and systematic prediction error (SPE) compare simulated with measured data. The number of all internodes above rank 4, which had reached final lengths are shown in column “Number.” Mean data of internode lengths, standard deviations and coefficients of variation comprise all internodes above rank 4, which had reached their final lengths at the day of measurement (DOY) or, in case of the simulation results, at the corresponding simulation step*.

### Prevailing and rank-specific PAR and temperature conditions used in both model approaches

The pattern of FILs and growing internodes along the main stem emerges from the performance of the individual internodes. In the model, this performance mainly depended on the rank-specific light and temperature conditions. Moreover, the rank-specific conditions are determined by the timing of internode appearances. Therefore, in the following, we take a closer look at the prevailing and rank-specific PAR and temperature conditions used in both model approaches (Figures 3, 5).

Using MA-T, the simulated durations between the appearance of two successive leaves varied between 1.0 d and 1.4 d for both experiments. The average durations were slightly shorter for E2. In contrast, this duration was constantly equal to 1.4 d with MA-20. Thus, in comparison with this temperature-insensitive approach, using MA-T resulted for these experiments finally in an up to 5 days earlier appearance of internodes at higher ranks. e.g., using MA-T the internode at rank 10 in E2 appeared 4 days earlier than with MA-20. This temperature-sensitive timing of appearances significantly influenced the rank-specific environmental conditions (Figures 3B,C, 4B,C). For E1, PAR4d (averages of daily mean PAR of 4 days starting 6 days before the internode appeared in the model) for ranks 8–13 and 18–20 were different for MA-T and MA-20. However, the time shift did not alter the PAR4d conditions for the internodes at the remaining ranks (Figure 3B). Due to the prevailing PAR-conditions for E2, PAR4d relevant for the ranks 6, 9, 12, 15 were similar for MA-T and MA-20, while the time shift changed the PAR4d conditions for the internodes at the remaining ranks (Figure 5B). For neither experiment, the temperature-dependent shift in appearance rates resulted in a correlation between rank-specific PAR4d and T4d data (*p* > 0.60).

### PAR and temperature contributions to average internode lengths

The average PAR during the experimental phase of E1 was 15% higher than in case of E2 (464 ± 116 μmol m^−2^ s^−1^ and 404 ± 120 μmol m^−2^ s^−1^, respectively). The average rank-specific PAR4d for rank 5–20 using MA-T was almost equal for E1 and E2 (375 ± 44 μmol m^−2^ s^−1^ and 383 ± 75 μmol m^−2^ s^−1^, respectively). Thus, the realized rank-specific PAR4d conditions resulted in an estimation of ca. 8.0 cm for the PAR-dependant internode length contribution for both experiments. In contrast to the PAR data, the average daily temperatures during the experimental phases were almost equal (21.9°C and 22.4°C). The average of the T4d values for rank 5–20 was 7% and was lower in E1 than in E2 (21.2°C and 22.8°C). The T4d data resulted on average in Arrhenius-factors *F*_av_(21.2) = 1.12 for E1 and *F*_av_(22.8) = 1.29 for E2. These factors correspond to temperature-dependant internode length contributions, i.e., an increase in the elongation rates of ca. 12 and 29% on average and in a decrease of the average elongation duration by 11 and 21% for E1 and E2, respectively. Finally, the temperature-sensitive model approach led to an average FIL of 9.4 cm for E1 and 10.4 cm for E2 (Table 1). This means that the difference of ca. +1°C in E1 and +3°C in E2 in the model-input temperatures (MA-T minus MA-20) plus emerging effects from R:FR ratios caused an overall increase of predicted mean FILs using MA-20 by ca. 1.4 cm in E1 and by ca. 2.2 cm in E2.

### PAR and temperature contributions to individual internode lengths

The temperature-sensitive model approach, MA-T mimicked the different patterns of individual internode lengths along the main stem for all measurements in both experiments very well (Figures 2, 4). Differences with measured data occurred for both experiments only at very few ranks. Therefore, we used model comparisons to assigning PAR × temperature contributions to individual internode lengths.

Scenario-based differences in individual FILs were up to 3.3 cm for both experiments. For example in E1, the highest differences occurred at ranks 5 and 12. At rank 5, small differences in PAR4d (PAR4d used in MA-T minus PAR4d used in MA-20) did not significantly contribute to the differences in FILs (Figure 3B). At this low rank, simulated R:FR ratios were not reduced. Therefore, temperature must have been the main contributor to differences in predicted FIL. In contrast, at rank 12, changes in PAR4d due to the temperature-induced differences in organ appearance already accounted for almost 60% of the differences in FILs (Figure 3B). Predicted FILs at the same rank were similar (<0.5 cm) at different combinations of PAR and temperature conditions. Here, the differences between the rank-specific PAR4d in MA-T and in MA-20 (<55 μmol m^−2^ s^−1^) and the corresponding differences in temperature (<1.3°C) were small (ranks 7, 8, 17, 18). Medium differences in predicted FILs of ca. 1.7 cm were either related to higher differences in PAR4d (rank 10, 55%) or to lower differences in PAR4d (rank 13, 23%). In addition, corresponding differences in T4d were three times higher at rank 13 (2.1°C) than at rank 10 (0.7°C) (Figures 3B,C). For E2, similar contribution patterns emerged (Figures 5B,C).

## Discussion

Nowadays, there is an increasing interest in analyzing the combined effects of two or more environmental factors on plant performance (Suzuki et al., 2014). To disentangle the effects of one environmental factor from the other is, to some extent, experimentally difficult and laborious. For example, under field and greenhouse conditions, changes in environmental factors may occur at the same time or correlate to each other. Moreover, under controlled conditions, e.g., in a plant growth chamber, space and accordingly number of possible repetitions are often very limited. Here, we analyzed the interplay between light, temperature effects, and final internode length of cucumber plants by modeling the effects on physiological processes. We found marked differences in the prediction qualities between the temperature-sensitive and the temperature-insensitive model approaches (Table 1). As expected, the temperature-sensitive model approach resulted in accurate mean internode lengths and number of internodes for both experiments and all time steps (Table 1). In the following, we discuss the three major insights gained by our study, which concern (i) the significance of average environmental data during experimental phases, (ii) the interplay between PAR, temperature and internode performance, and (iii) the validity of the temperature response model approach.

Our analyses revealed interesting patterns of obtained average climatic data during the experimental phases in relation to the corresponding average rank-specific data realized using the temperature sensitive model. While for the PAR-data the averages differed for the two experimental phases and were similar for the rank-specific data, there were almost no differences in the average daily mean temperatures during both experimental phases, but the average realized rank-specific temperatures differed. Thus, using the average environmental data during the experimental phases in our model approach we might have concluded that PAR played a major role in the experiment-specific differences of average final internode lengths, but temperature did not. Yet, from the rank-specific realized data, a different picture emerged: Temperatures played an important role, but not PAR. Thus, this discrepancy between average environmental conditions during experimental phases and average rank-specific conditions highlights the choice of respective data in order to assess the effect of environmental conditions on internode length. This assessment is, for example, of particular interest for the construction dose-response curves by means of meta-studies (Poorter et al., 2010).

In the two experiments of this study, the temperature effect on appearance rates resulted in different patterns of time shifts and, consequently, different experiment-specific patterns of PAR signals and temperature effects for internode growth (Figures 3B,C, 5B,C). For both conditions, the temperature-sensitive model approach predicted the pattern of final internode length along the main stem very well (Figures 2, 4). The detailed model analysis based on model comparisons revealed that various combinations of realized PAR and temperature led to similar internode lengths. At some ranks, realized model-induced differences in temperature conditions compensated differences in PAR conditions, which were induced by the temperature effect on the timing of internode appearance. Therefore, the model comparisons highlight that the effective light signals perceived by an internode during its developmental stage can be influenced by temperature through its effect on internode appearance rate. This might also explain the rank-shifts between measured and simulated data in the previous study of Kahlen and Stützel (2011a), where the temperature effects are not considered. Several other studies provide further details about the light and temperature signal crosstalk in plant development (e.g., Franklin, 2009, Koini et al., 2009). Here, we demonstrated that cucumber, a crop highly sensitive to changes in the environment, might be an appropriate model crop for up-scaling knowledge gain from the organ to canopy level.

Despite marked effects of the temperature conditions on internode appearance in both experiments, the rank-specific temperatures used for model predictions with the temperature-sensitive model approach covered only a range from 20°C to 24°C. Thus, even if daily mean temperatures vary within a small range of a few degrees, such as in typical greenhouse production systems, for reliable model predictions the temperature-response of internodes in terms of appearance rates and growth rates have to be taken into account. On the other hand, from our study it remained unclear how the temperature-sensitive model approach would have performed under temperature conditions below 20°C and above 24°C. Moreover, some deviations between measured and simulated internode lengths using the temperature-sensitive model approach might be related to the rough parameter estimation for the Arrhenius function. Thus, for a wider range of applications using this model approach, a sound crop-specific temperature response function would be of importance.

Here we would like to point out several aspects in modeling temperature effects on the plant development and growth. First, a crop-specific parameterization of the Arrhenius function could be based on meristem temperatures (e.g., Parent and Tardieu, 2012) or air temperatures (Kang et al., 2012; and this study). A recent study (e.g., Savvides et al., 2013) showed systematic deviations of up to ± 4°C between air temperature and meristem temperature for cucumber plants. These deviations were explained by the influence of other environmental variables (e.g., vapor pressure deficit, radiation, and wind speed) on the transpiration rate of the young unfolded leaves covering the meristem. It is possible that, as concluded by Savvides et al. (2013), a model shift from air temperature to meristem temperature could improve the prediction quality of the temperature sensitive model. However, this spatial shift in temperature should be in line with a temporal shift, i.e., the shift from appearance rates to initiation rates. However, whether organ appearance rates would be more related to meristem temperature, influencing developmental processes, or to air temperature still requires further examination. Accordingly, it would be useful to develop a model for predicting meristem temperature based on air temperature or other environmental factors, if a model should predict leaf initiation instead of leaf appearance rates. Second, our model simply assumed that appearance rates and organ aging are driven by the current daily mean temperatures, while elongation rates depend on the mean temperatures of 4 days starting 6 days before the internode appeared in the model. However, as in monocotyledon plants, actual temperature during organ growth might also influence internode performance (e.g., Poiré et al., 2010). Thus, there is still need for a systematic analysis of the timing and duration of the temperature sensitive time window for all developmental processes, such as elongation rates and phyllochron. Third, in greenhouse production systems, the settings for day and night temperatures can be used to control stem lengths. Differences in day/night temperatures (DIF) have as well an effect on average internode length of cucumber (de Koning, 1992). Thus, the chosen model approach would not be able to mimic DIF-driven changes in internode growth patterns. Last, but not least, there is also a need to discuss the chosen model approach itself. Alternatives to the Arrhenius function for the developmental response to temperature do exist. A recent paper on predicting maize phenology evaluated, inter alia, the precision of eight thermal functions (Kumudini et al., 2014). The Arrhenius approach was classified as a process-based function and was identified as most suitable for high, supra-optimal temperatures. To support this point, further experimental data over a wide range of temperature conditions are needed.

In summary, simulations performed in this study showed the interplay of temperature and light conditions on internode performance of cucumber plants. The detailed analysis clearly suggests that the model containing both, light, and temperature, as predictors is superior to the model that only contains the predictor light. Interestingly, average environmental data during experiments may not allow accurate predictions of average contributions of environmental factors on internode performance. Furthermore, there is still need for a proper cucumber-specific parameterization of the Arrhenius function for a wide range of temperature conditions to open avenues for future research on plant performance under changing environmental conditions. Possible applications of the improved model could be, e.g., for better controlling climatic conditions in greenhouse production systems of cucumber plants or for assessing climate change impacts on the performance of field-grown crops.

### Conflict of interest statement

The authors declare that the research was conducted in the absence of any commercial or financial relationships that could be construed as a potential conflict of interest.
