# Supplementary material for: Predicting Plant Performance Under Simultaneously Changing Environmental Conditions—The Interplay Between Temperature, Light, and Internode Growth
Source: Front Plant Sci. 2015 Dec 21;6:1130. doi: 10.3389/fpls.2015.01130 (PMC4685136; doi:10.3389/fpls.2015.01130)
Supplement: Supplementary file 1 [file Table1.pdf]

Supplementary Material

Predicting plant performance under simultaneously changing environmental conditions – the interplay between temperature, light and internode growth

Katrin Kahlen[1]*, Tsu-Wei Chen[2]

[1]Department of Vegetable Crops, Geisenheim University, Geisenheim, Germany
[2]Institute of Horticultural Production Systems, Leibniz Universität Hannover, Hannover, Germany

*** Correspondence:** Katrin Kahlen, Department of Vegetable Crops, Geisenheim University, Von-Lade-Straße 1, 65366 Geisenheim.
Katrin.Kahlen@hs-gm.de

1.1. Supplementary Tables

Supplementary Table 1. Time schedule for the two experiments, E1 and E2.

	E1	E2
Sowing	01.06.2012	26.03.2014
Transplanting	14.06.2012	10.04.2014
Start treatments	21.06.2012	14.04.2014
